# Policy in practice: assessing Senegal’s family planning progress using a mixed-methods approach

**DOI:** 10.1136/bmjgh-2024-018774

**Published:** 2026-06-09

**Authors:** Sylvain Landry Birane Faye, Georgette Helene Coumba Sow, Rose André Faye, Marie Gabrielle Ndong, Martine Eva Tine, Ndeye Awa Diagne, Amadou Doucoure, Hina Najmi, Mishal Zulfiqar, Sacha St-Onge Ahmad, Zahid Ali Memon, Zulfiqar Bhutta

**Affiliations:** 1 Laboratoire de Sociologie, d’Anthropologie et de Psychologie (LASAP- ETHOS) FLSH, Universite Cheikh Anta Diop de Dakar, Dakar, Dakar Region, Senegal; 2 S&F Pro Consulting LLC, Manteca, California, USA; 3 Mother and Child Health Directorate, Government of Senegal Ministry of Health and Social Action, Dakar, Dakar Region, Senegal; 4 Institute of Global Health and Development, The Aga Khan University, Karachi, Sindh, Pakistan; 5 The Hospital for Sick Children, Toronto, Ontario, Canada; 6 Community Health Sciences Department, The Aga Khan University, Karachi, Sindh, Pakistan

**Keywords:** Africa South of the Sahara, Global Health, Health policy, Maternal health, Interdisciplinary Research

## Abstract

**Background:**

Senegal has made significant progress in family planning (FP), with the modern contraceptive prevalence rate (mCPR) increasing from 12% in 2010 to 26% in 2023, along with a sharp decline in unmet need. Once among West Africa’s lowest performers, Senegal now stands out for its advancements in FP access and equity.

**Objectives:**

This study examines the main factors driving Senegal’s FP progress—including policy leadership, financing mechanisms and community engagement—while identifying ongoing challenges and lessons for policy and practice.

**Methods:**

We employed a mixed-methods approach, combining trend and decomposition analyses with a review of FP policies, financing and programmes, guided by the WHO’s health system building blocks. Qualitative data were collected from interviews and focus groups with policymakers, providers, community leaders and users across various regions.

**Results:**

Senegal’s early gains in FP were driven by strong political leadership, task-shifting, community-based service delivery and supply chain improvements. Social mobilisation campaigns, including *Moytou Nef* and engagement with religious leaders and men, promoted supportive norms and increased demand. These efforts improved method availability, reduced stockouts and expanded access. Decomposition analysis identified FP knowledge (35%), education (18%) and facility access (16%), particularly among young women, as the main drivers of performance. Broader shifts in gender norms, women’s empowerment and male involvement also supported uptake. Since 2017, mCPR has plateaued, with persistent gaps among adolescents, unmarried women and rural populations due to stigma, provider bias, limited youth-friendly services and donor dependence. These findings highlight the need for targeted, equity-focused interventions to sustain and expand Senegal’s FP achievements.

**Conclusion:**

Senegal’s FP progress demonstrates how strategic governance, service innovation, and community engagement can increase contraceptive use. To sustain and build on these achievements, renewed investment is necessary in youth-focused services, domestic funding, and equity-centered strategies that are integral to larger health and development plans.

**WHAT IS ALREADY KNOWN ABOUT THIS TOPIC**

- Senegal is recognised as a rare family planning (FP) success story in West Africa, with modern contraceptive use rising steadily from a low baseline.
- Most prior studies have focused on donor-funded programmes and their outcomes, overlooking the role of sustained national leadership, strong governance and coordinated policy action in enabling this progress.
- Despite clear gains, deep and persistent inequities—especially among adolescents, rural women and conservative communities—continue to threaten equitable FP access.

**WHAT THIS STUDY ADDS**

- Provides the first comprehensive analysis of Senegal’s FP progress, revealing how deliberate policy sequencing, political consistency and innovation in service delivery and supply systems sustained long-term improvements.
- It demonstrates that institutional continuity, adaptive governance and community-based models were crucial in expanding access and resilience across regions.
- Demonstrates that gender-transformative action—not male engagement alone—is essential to advance women’s autonomy and tackle entrenched social norms limiting FP use.

**HOW THIS STUDY MAY AFFECT RESEARCH, PRACTICE OR POLICY**

- Offers a replicable mixed-methods framework for assessing reproductive health progress in low- and middle-income settings.
- Strengthens the case for embedding FP within broader health system reforms, with sustained investment in trained community health workers and decentralised services.
- Calls for a shift from short-term awareness campaigns to long-term, culturally grounded norm-change strategies led by trusted community actors and backed by political and financial commitment.

## Introduction

 The WHO emphasises meeting contraceptive needs and expanding access as essential to achieving universal sexual and reproductive health.^1^ The 2030 Agenda^2^ has intensified efforts to strengthen family planning (FP) in sub-Saharan Africa,^3^ which is vital for maternal and child health,^4^ return-on-investment strategies^5^ and poverty reduction.^6^ Demographic Health Survey (DHS) data show child mortality declined from 51 per 1000 live births in 2016 to 37 in 2019. The fertility rate decreased from 6.0 in 1992 to 4.3 in 2022, reflecting current trends.^7^ Senegal reduced maternal mortality from 392 per 100 000 in 2011 to 153 in 2023. The WHO and World Bank estimates in 2023 place the MMR at ~237, which is below the regional average.^8^

While FP access remains difficult in West Africa,^9^ Senegal’s modern contraceptive prevalence rate (mCPR) grew from 7.6% in 2005 to 17.9% in 2019 and 25.6% in 2023, a significant achievement for a country with the lowest rate for years.^10^ The government also reported increased demand for FP with modern methods (mDFPS), from 38% in 2012 to over 55% in 2022. Compared with neighbours like Mali (34%) and Guinea (36%) in 2018, Burkina Faso (48%) and Côte d’Ivoire (44%) in 2021, Senegal’s mDFPS progress is rapid. Despite challenges in equity, adolescent access and regional disparities,^11^ Senegal’s FP progress shows strong government commitment, as in the Senegal Emerging Plan.^12^

Senegal’s progress has earned recognition as a West African model by the Exemplars in Global Health (EGH), supported by Gates Ventures. This initiative identifies successful outlier countries in global health, analyses their success factors and shares lessons for policy. Senegal, with five others, was selected based on steady mCPR and demand improvements, adjusted for development using the Human Development Index. Other analyses, using gross domestic product and compound annual growth rate (CAGR), confirmed its high ranking based on Institute for Health Metrics and Evaluation data. Selection also considered geographic diversity and research feasibility. Senegal provides insights into FP progress amid sociocultural challenges like resistance from religious leaders.^13^ Despite recent data showing a slowdown in mCPR growth since 2017, ongoing support persists. The rapid increase in the under-35 population^14^ hampers efforts to expand FP services and adolescent reproductive health, highlighting the need to review achievements, gaps and challenges to sustain and accelerate FP progress.

Despite Senegal’s progress, the literature primarily focuses on programmatic successes and headline indicators such as mCPR, neglecting a detailed exploration of where, for whom, how or why progress occurs, as well as the drivers or gaps.^15^ FP performance is assessed via mCPR, unmet need and demand satisfied—indicators of access, use and autonomy. While useful, mCPR may not fully reflect fertility intentions or service quality. Progress can happen through decreased unmet need or increased demand satisfied, even if mCPR remains stable, especially as fertility preferences change. Most studies overlook political, cultural, systemic or governance factors,^16^ rarely combining quantitative trends with qualitative insights on policy and funding.^17^ Recent stagnation, youth access barriers and disparities are underexplored.^18^ This study employs a mixed-methods approach with four data sources to examine Senegal’s FP trajectory (2010–2022), analysing mCPR, unmet need and demand satisfied for an equity-focused view amid changing norms and models. Triangulating demographic, policy, financial, qualitative and household data provides insights into progress and challenges, informing policy, programming and research.

## Methodology

### Study settings

Senegal, a West African lower-middle-income country (LMIC) with 18.1 million people, has a young population—about 60% under 25 years—and a dependency ratio of 75.2%. Despite a 2.9% growth rate (2013–2023), fertility rates have decreased by 1.5 children per woman since the early 1990s. FP is vital for demographic dividends and sustainable development. Adolescent fertility is a concern, with 14% of girls aged 15–19 years having begun childbearing.^14^

Senegal has 14 regions, 79 health districts, 110 health centres, 1531 health posts and 2283 health huts. Health centres and health posts offer *all long-acting reversible methods*, as well as short‐acting methods; health huts provide male and female condoms, contraceptive pills and injectables.^19^ In 2012, Senegal’s public health facilities generally had high availability of contraceptives, with over 90% stocking methods like intrauterine devices (IUDs) (94.6%), male condoms (92.7%), injectables (92.6%), oral pills (89.5%) and others.^20^ However, stock-out rates were high, with injectables unavailable about 43% of the year and implants roughly 83%.^21^,^9^ By 2022, approximately 90% of service points had not experienced stockouts in 6 months.^22^ Use has increased over the past decade; however, disparities persist. FP now expands beyond married women aged 15–49 years to include adolescents and unmarried youth, despite cultural sensitivities.

Cultural and religious contexts play a strong role in shaping reproductive behaviours. Approximately 95% of Senegal’s population is Muslim, and Islamic teachings significantly influence social norms and attitudes towards contraception. Qualitative studies found that social-norms and influential community and religious actors shape women’s contraceptive decision-making in four Senegalese regions.^23^ The strategy now includes task-shifting to community health workers (CHWs), engaging religious leaders, mobile outreach and supply chain improvements.

### Data sources

This study used a mixed-methods approach based on the EGH framework, comprising four parts. Quantitative data focused on contraceptive use and attitudes; qualitative data examined environmental and political contexts, stakeholders’ perceptions, practices, facilitators and barriers.

*A systematic review* examined FP financing, policy and progress in Senegal through 2022, following Preferred Reporting Items for Systematic Reviews and Meta-Analyses (PRISMA) 2020 guidelines. Evidence was gathered from peer-reviewed articles, reports and grey literature through PubMed and Web of Science for studies published between January 2000 and December 2022 (see online supplemental table 1). Keywords included “family planning,” “contraception,” “financing,” “policy” and “Senegal.” Reference lists from included papers and reviews were checked for additional sources. Two reviewers independently screened titles, abstracts and full texts, resolving disagreements through discussion. Included studies provided data on FP outcomes, financing or policy; editorials and unrelated publications were excluded. Data were extracted using a standardised template that covered study details, FP indicators (such as mCPR and demand satisfaction), financing and policy components, strategies and results. A thematic synthesis identified facilitators and barriers to FP progress, such as political commitment, community engagement, service access, contraceptive availability, governance and technological innovation. Quantitative trends in mCPR and demand satisfaction supported the thematic findings.

*Quantitative analysis* used the DHS (1986–2019) and United Nations data (1990–2020) to identify trends in mCPR and demand by wealth, residence and education. mCPR measures women aged 15–49 years using modern contraception at survey time. The Oaxaca-Blinder method distinguished between demographic effects and behavioural changes, clarifying whether mCPR improvements resulted from demographic shifts or increased use within groups. DHS data from 2005 to 2019 supported six subgroup models based on age and marital status for women aged 15–49 years, including those sexually active in the past 12 months, not pregnant or menopausal and fecund (classified via self-reports on fertility, menstrual history, contraceptive use and childbearing). Variables were analysed at individual and ecological levels, controlling for region, religion, age, ethnicity and survey year across five macro-areas.

*The policy, programme and financing review* (PPFR) analysed Senegal’s FP landscape from 2000 to 2022 using WHO health system building blocks. It reviewed policies, plans, financial data and evaluations, including frameworks, Ministry of Health and Social Action (MoHSA) reports, donor agreements and initiatives like community-based distribution and youth programmes. The review focused on governance, coordination and financial sustainability within broader frameworks of gender, youth and reproductive rights. A timeline linked the evolution of FP policy with mCPR trends and demand. Financial analysis covered government, donor and out-of-pocket funding, based on evaluations of the National Family Planning Action Plan (NFPAP, 2010–2015) and the Strategic Framework (2016–2020). It assessed spending priorities, policy alignment and linked data with FP outcomes. The PPFR identified enablers like policy coherence and innovation, along with challenges in equity, coverage and financing affecting FP in Senegal.

*A qualitative study* examined Senegal’s FP performance in six districts chosen for variation in geography, culture and FP outcomes based on national data. Although not statistically representative, the selection aimed to highlight key differences. Participants were purposefully selected to represent diverse perspectives on FP, considering demographics, roles and community influence. Semistructured interviews (SSIs) with 30 policymakers, 79 health workers, CHWs and 66 FP beneficiaries examined perceptions, practices and institutional views on FP. Interview guides were tailored, tested and refined for Senegal. Focus group discussions (FGDs) with adolescent boys and girls, married and unmarried adults and health workers explored community norms. Additionally, 120 household observations were conducted where at least one woman of reproductive age (WRA) 15–49 years lived, assessing influences on FP decisions—focusing on negotiation processes, women’s autonomy, CHW interactions and sociocultural norms. These households were intentionally selected to ensure geographic diversity, varied service delivery settings and community engagement experiences. All discussions and observations were conducted in local languages (Wolof, Pulaar, Diola or French) by 12 trained researchers with backgrounds in social sciences and public health. Verbal or written consent was obtained, including assent and parental permission for minors. All sessions were audio recorded, transcribed, anonymised and stored securely. Thematic content analysis was performed using ATLAS.ti. A coding framework was developed deductively based on the study objectives and refined inductively through iterative review of transcripts. Triangulating data across SSIs, FGDs and observations allowed for the identification of recurring themes, differences and underlying values. While the findings are not generalisable, purposive sampling and triangulation improved contextual validity. Limitations include potential social desirability bias and translation loss.

Findings from the four research components were integrated through a structured convergence process combining quantitative and qualitative evidence. Quantitative results on mCPR and demand satisfaction were triangulated with qualitative insights on policy implementation, social norms and service delivery. A four-phase synthesis—encompassing preliminary analysis, internal validation, stakeholder consultation and final contextual refinement—ensured coherence across data sources, enhanced validity and grounded interpretations in local evidence and perspectives.

## Results

### Overview of mCPR and demand-satisfied national progress

Between 2010 and 2023, Senegal saw significant changes in FP indicators. The mCPR among married women rose from 12.0% in 2010^24^ to 26.3% in 2017, then slightly declined to 25.6% in 2023,^25^ despite overall long-term growth, especially among sexually active unmarried women at about 40%. The CAGR was 2.9% (1997–2011), then 9.8% (2012–2019), averaging about 1% from 2014 to 2019.^26^ D*emand for modern methods* increased from 38.2% in 2010 to 63.2% in 2019, plateauing at 58–60% by 2023. Gains were greater among urban, educated, wealthier women; rural women and adolescents faced barriers. U*nmet need* decreased from 30.1% in 2010 to 19.1% in 2023, with urban and wealthier women progressing faster than adolescents and unmarried women.

**Figure 1:**
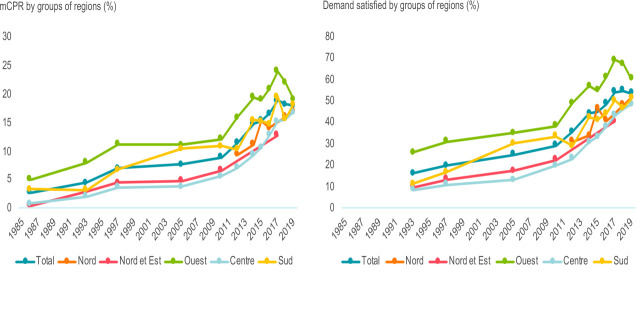
Regional differentials in modern contraceptive prevalence and demand for family planning satisfied in Senegal, by zone (1985–2019). mCPR, modern contraceptive prevalence ratemodern Contraceptive Prevalence Rate.

DHS data from 2010 to 2019 show that regional disparities decreased since 2017, with the western and central regions leading in mCPR and FP demand satisfaction, while the eastern and northern regions lag (figure 1).

**Figure 2:**
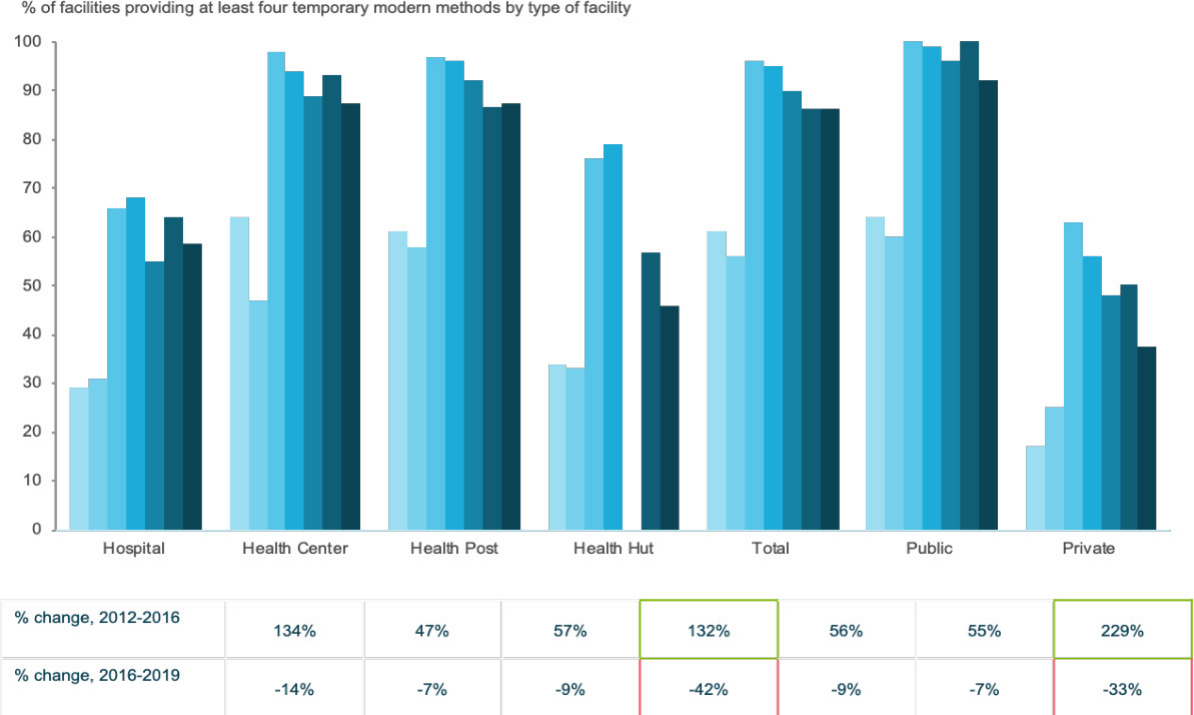
Availability of contraceptive methods by health facility type in Senegal (2012–2019).

By 2019, Dakar, Thiès and Ziguinchor had the highest mCPR among married women, over 65% of FP demand met, reflecting stronger health systems, urban health services and ongoing investments. In contrast, Kolda, Kédougou, Tambacounda and Matam (East and North) had low mCPR (often below 15%) and demand satisfaction below 45%, due to weak service delivery and sociocultural barriers.

Between 2005 and 2023, Senegal improved FP equity, with the urban–rural mCPR gap shrinking from 5.5 to 2 percentage points and rising across all wealth groups due to pro-poor policies. However, access remains unequal for young women, especially adolescents, who face slow progress. DHS data show adolescents lag in mCPR, demand, and unmet need, with only about 3% increase among 15–19 year-olds in 2017. Sexually active, unmarried adolescents had about 24% usage, highlighting hidden disparities.

The low mCPR among adolescents might hide progress: demand for modern methods rose from 49% to 60%, and unmet need fell from 31% to 25% between 2010 and 2020. Unmarried and rural teens often face stigma and lack access to youth-friendly services. Gender norms and legal restrictions mean adolescent mCPR does not fully reflect reproductive autonomy, access or contraceptive needs. To better understand FP progress, key indicators like demand satisfaction, method mix and equity data must be considered.

Between 2010 and 2017, Senegal expanded contraceptive options, including long-acting methods alongside injectables. In 2010, short-acting methods dominated, with injectables over 60% among married women and long-acting under 5%.^27^ Access improved, with implant use sharply rising to 20–25% by 2019, and mCPR increasing from 20.3% in 2014 to 25.5% in 2017. By 2023, injectables remained common at 45–50%, with implants growing, especially in rural areas, for their convenience.

DHS data show regional differences in contraceptive use, with injectables most common in Fatick (50%) and Matam (59%) due to availability through health huts and CHWs. Many women switch from short-acting to long-acting methods, indicating increased access and trust,^28^ with regional preferences varying. In rural, conservative areas with high male emigration and spousal absence, discreet long-term contraceptives are valued for effectiveness and convenience. However, a multilevel analysis shows Kolda has many FP facilities, but only 22% offer all methods.^29^ This discrepancy raises questions about supply chain issues and sociocultural barriers that may restrict method mix availability at the facility level.

### Assessing the main factors behind Senegal’s FP progress

#### Drivers of FP progress: evidence from Oaxaca-Blinder Decomposition

The Oaxaca-Blinder decomposition table (table 1) presents the results of an analysis examining the factors contributing to changes in mCPR among women aged 15–49 years in Senegal between 2005 and 2019. It shows a 15.2-point rise in mCPR from 2005 to 2019, with 35% explained by the model. Most gains came from knowledge, education and access, making up nearly 70%. The most significant factor (35%) was increased understanding of modern methods, highlighting successful demand-generation efforts; while education contributed 18%. Access, based on health visits, accounted for 16%, focusing on routine services and outreach. Changing gender norms, such as rejecting wife-beating, explained 14%, showing norm change influences contraceptive behaviour alongside supply. Conversely, delayed sexual debut was linked to an 11% decrease, possibly due to less immediate need among teens starting sex later.

**Table 1:** Oaxaca-Blinder decomposition of changes in modern contraceptive prevalence rate (mCPR) in Senegal, 2005–2019 (DHS)

Factor	Contribution to mCPR change (%)	Interpretation
Modern FP knowledge	35%	Reflects the impact of awareness campaigns and educational programmes on contraceptive uptake.
Education level	18%	Increased schooling improved women’s autonomy and ability to make informed FP choices.
Health facility visits	16%	Improved access to care enabled more frequent FP counselling and service delivery.
Social norms (eg, wife-beating rejection)	14%	Shifting gender norms, especially around agency and decision-making in relationships.
Household wealth	5%	Modest gains from improved socioeconomic conditions among lower-income groups.
Age at first sex	–11%	Delayed sexual debut may reduce immediate need for contraception, reflecting nuanced fertility-control dynamics.
Other factors	3%	Includes media exposure, marital status and employment variables.

DHS, Demographic Health Survey; FP, family planning.

These results highlight the complex relationship between fertility preferences and contraceptive use, especially among adolescents, where biases and hidden needs may affect measurements. Senegal’s FP progress was driven by increased knowledge, access and changing gender norms, supported by political commitment. These efforts, backed by public-private partnerships and health funding, led to a rapid rise in mCPR from 2012 to 2017, linked to the 2010 Assistance for Health program, health sector reforms by international partners (United States Agency for International Development (USAID)), the World Bank, United Nations Population Fund and bilateral donors, Senegal’s FP2020 commitment and supply chain improvements.

#### Key political moments and interventions relate to mCPR and demand progress

Senegal’s progress in contraceptive use and demand fulfilment is closely tied to political milestones (online supplemental table 2). *Adopting international policies an*d key laws from the 1980s shows strong FP political commitment. The 1992 Bamako Initiative influenced health policy across sub-Saharan Africa, shaping Senegal’s FP through decentralisation and privatisation reforms.^30^ After contraception was legalised in 1980,^31^ the 1988 Population Policy saw FP as a tool to curb population growth,^32^ leading to the 1990 national FP programme. Integrated into the Directorate of Reproductive Health in 1998, FP gained momentum with the 2002 Population Policy revision and the 2005 Reproductive Health Law, emphasising equitable access, especially for disadvantaged women. This shift moved from population control to a rights-based, equity focus. Early efforts aimed at demographic goals, but later reforms embedded FP in broader reproductive health strategies, promoting inclusive, demand-driven approaches after 2010. In 2011, Senegal endorsed the Ouagadougou Call to Action and pledged to increase FP funding, with MoHSA leadership crucial in meeting demand and implementing reforms.

FP services began in Senegal in 1964, with the 1990 Population Policy marking the launch of a national FP program. The creation of the National Service of Reproductive Health strengthened coordination and policy development. Since the London Summit, the government has reaffirmed its commitment through budgeted action plans and supportive policies, including the Law on Reproductive Health, the PSE [Plan Sénégal Émergent], and the National Health Development Plan. Strong national leadership has been key to repositioning FP as a development priority*—*former Head of the DPF Division.

At the 2012 London Summit on FP, Senegal pledged to double health investment and expand FP access. This resulted in the NFPAP 2012–2015,^33^ combining increased funding with a rights-based approach. The three-dimensional model—democratisation, decentralisation and demedicalisation—helped address barriers by empowering communities, healthcare workers and users, boosting contraceptive use in underserved areas^34^ and paving the way for more equitable FP services, as later frameworks 2016–2020 emphasised reproductive rights and cross-sector collaboration.^35^

*Senegal’s FP progress relies on governance, decentralisation and multisectoral cooperation*. Since 1997, MoHSA institutionalised leadership via directorates. The Reproductive Health and Child Survival Directorate, created in 2002 and upgraded in 2012, oversaw FP. A technical committee coordinated partners, donors and civil society. In 2016, the Directorate of Maternal and Child Health established a multisectoral framework involving ministries, civil society and partners, focusing on access, demand and contraceptive security. The FP Task Force and Dialogue Frameworks aligned policies, mobilised resources and coordinated efforts through meetings that shared data, set priorities and engaged in advocacy.^36^ Responsibilities shifted to regional and district health teams, enabling locally tailored FP strategies. This framework allowed community-responsive FP strategies addressing supply-side and demand-side challenges, essential for equitable progress and continuity through political and administrative changes.

Since 2005, Senegal has used District Health Information Software 2 for *real-time health data, enhancing planning and interoperability*. In 2009, MoHSA created the Division of Studies and Health Research to coordinate efforts. Regular donor meetings aligned data use with policy. Since 2011, MoHSA and Agence Nationale de la Statistique et de la Démographie have conducted annual DHS surveys to guide programmes and monitor key indicators. The 2012 NFPAP emphasised research and university partnerships. Investing in data systems and collaborations supported evidence-based decisions, strengthening health planning, policy and accountability. However, data gaps in the private sector and local authorities highlight the need for decentralisation and inclusive decision-making.

Senegal’s political leadership and strategies fostered a supportive policy environment, improving access to services, supply chains, outreach and equity. These reforms increased modern contraceptive use from 12% in 2010 to 25.5% in 2017, with demand satisfaction over 63% by 2019.

#### Financial commitments and donor support: Factors influencing mCPR growth and recent stagnation

*Senegal’s post-2012 financial commitments showed a move towards domestic control of its FP agenda*. The government pledged a 200% increase in contraceptive procurement funding and doubled its contribution to the NFPAP.^37^ However, FP programmes rely heavily on external funding, risking shifts in donor priorities. Senegal received support from the USA and the Gates Foundation, boosting its capacity. From 2012 to 2015, US$46.8 million was mobilised for FP, mainly for commodities. The government doubled its contribution to US$1.15 million (2.5%), while donors funded 89.5%: USAID (57.5%), Gates Foundation (21.3%) and UNFPA (16.1%). Investments improved supply chains and mCPR from 2012 to 2017 through commitments, scale-up and community models. Policies like price cuts and tax exemptions increased affordability, positively correlating with mCPR growth (r=0.83). In 2017, Senegal pledged to increase its contraceptive budget from US$477 000 (2016) to 795 000 by 2020. US$69.4 million was mobilised, with 48.7% for commodities. Bilateral funding was 25.2%, private and multilateral funding declined. USAID’s share fell to 15% (2016) and 14% (2017), with unchanged mCPR and rising unmet needs.

*Domestic financing efforts:* as external funding declined, Senegal boosted local resource mobilisation for FP. The Challenge Initiative (TCI), funded by the Gates Foundation, encouraged local governments to take ownership of FP finances, leading 150 mayors to allocate funds for contraceptive supplies and establish reproductive health/FP budgets.^38^ NGO *Réseau Siggil Jigéen* mobilised over US$825 000 from municipalities for services like insurance and ambulances, improving access in remote areas. Public funding increased by 27% from 2016 to 2017, raising the government’s share from 27.6% to 39.2%.

Public financing has expanded FP access in Senegal by reducing costs and supporting sustainability. The government allocated a contraceptive budget, eliminated import taxes to lower prices and community programmes helped remove financial barriers, especially for low-income and rural women. Senegal aimed for 20% annual growth in domestic financing to boost contraceptive sovereignty, but the FP programme still relies heavily on external donors. Their continued support has been crucial; however, recent stagnation highlights vulnerabilities like reliance on external funds and disbursement delays, emphasising the need for increased local financing. Challenges include limited use of local budgets, low domestic funding (about US$0.45 per person) and inconsistent budget execution despite donor support.

#### Enhancement of FP’s supply chain and delivery services

Several targeted policies and interventions have helped build a resilient FP supply chain, close service gaps and support a more decentralised contraceptive delivery system. Since 2010, Senegal has *expanded contraceptive access through community efforts and legal reforms,* including task-shifting that allows nurses, CHWs and pharmacies to provide methods like IUDs, injectables and implants—mainly benefiting rural populations.^39 40^ The Senegal Urban Health Initiative increased modern contraceptive use from 10% in 2005 to 21.2% in 2015, with injectables available by 2017, and implants becoming the most used by 2019.^41^ Progress was also driven by provider training, improved supply chains and CHW outreach, reducing access gaps and attracting new users. CHWs, delivering counselling and services at health huts and outreach sites, helped change attitudes and boost adoption of long-acting methods in underserved areas.^42^

To address contraceptive stockouts, *Senegal’s MoHSA implemented the Informed Push Model (IPM) in 2011–2012 and expanded it by 2013*. IPM shifted from a passive ‘pull’ system to a proactive approach with private logistics providers delivering contraceptives monthly based on real-time data.^21^ Facilities received supplies on credit and paid only for sold stock. Within 6 months, stockouts fell below 2%, with shortages of implants and injectables decreasing by 83% and 43%, respectively. This led to a significant increase in contraceptive use—implants (+221%), injectables (+121%), IUDs (+68%), pills (+127%)—and an 11% rise in mCPR by 2014.^43^ In 2015, Senegal’s National Pharmaceutical Supply Agency (PNA) launched *Jegesi Naa* to decentralise procurement, improving access and reducing costs.^44^ It later became *Yeksi Naa*, integrating contraceptives into the essential medicines supply chain. By 2017, PNA had complete control. A policy requiring facilities to remit 25% of FP product revenue back to PNA led to financial strain, payment delays and stockouts of implants and injectables. The IPM initiative shows how public-private partnerships and real-time logistics reduce stockouts and increase contraceptive use. Yet, setbacks after shifting to national ownership reveal that maintaining supply chain resilience relies on technical reforms, long-term financial planning, institutional support and clear incentives.

*Senegal has enhanced its healthcare infrastructure for FP* through facility upgrades, workforce development and supply provision. Since 2006, the Community Health Program, supported by USAID, and the 2014 National Strategic Plan for Community Health have integrated community structures into the formal health system. To date, all districts are served by networks of health centres, posts, huts and outreach sites. Between 2012 and 2016, the number of FP service delivery points increased, especially in health huts and private facilities.^45^ Although service provision declined after 2017, this was partly offset by an increasing role for community-based and private-sector providers (figure 2).

**Figure 3:**
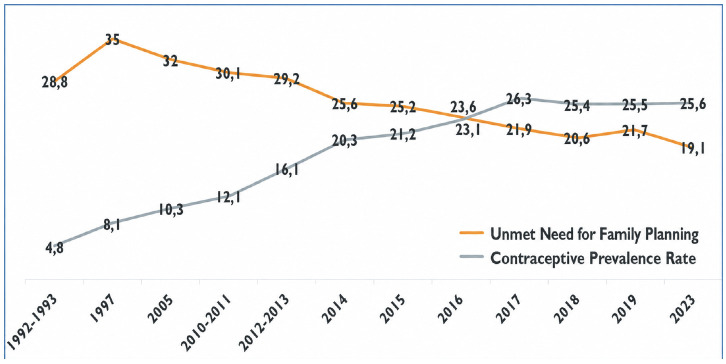
Trends in modern contraceptive use and unmet need for family planning in Senegal, 1992–2019 (Demographic and Health Surveys).

Human resource reforms, including incentives and training, cut provider caseloads by 42%, boosting service uptake and reducing clients’ travel time.^46^ The TutoratPlus programme trained health workers in counselling and service delivery.^39^ These efforts increased FP access, raising modern contraceptive use by 16 percentage points from 2012 to 2016, and narrowed the rural-urban gap from 11% in 1997 to 2% in 2019 (figure 3). Service coverage has expanded through community providers. Still, workforce and skill gaps remain, especially in rural areas. Although primary care and outreach have increased, youth-friendly and gender-sensitive counselling remain inconsistent, with stigma restricting access for adolescents and unmarried youth in rural regions.

*Community-based outreach and mobile delivery innovations:* since 2010, Senegal has launched community initiatives to improve access to contraceptives. Environnement et Développement du Tiers Monde (ENDA) Santé and Marie Stop International (MSI) used mobile clinics, initially for HIV, to deliver FP services in underserved areas. MSI created a social franchise for private providers to offer affordable implants and IUDs. The ‘Mobile Midwives’ programme involved CHWs organising outreach to promote quality FP in remote communities,^47^ raising awareness and demand through education. Supported by TCI, Senegal piloted FP Special Days at facilities and community sites. CHWs did door-to-door outreach; municipalities covered 35% of costs to offer free FP, supervised by nurses or midwives. This expanded reach and increased uptake among hard-to-reach groups.^48^

Supply-side innovations improved FP access, trust and quality. MSI’s mobile outreach mainly targeted rural and peri-urban women—41% were first-time users, and 39% chose long-acting methods like implants and IUDs.^49^ MSI trained private providers through its social franchise, expanding access and reducing reliance on pills and injectables. ENDA Santé’s mobile clinics provided FP, HIV and primary care in remote areas, overcoming transportation barriers. From 2012–2015, outreach accounted for 65% of new long-acting contraceptives, up from 47% at fixed sites. Continuation rates remained high: 99% for IUDs, 77% for injectables, but oral pill continuation was lower in outreach (65%) than fixed sites (84%).^50^

#### Demand creation policies: Raising awareness and shifting norms

*Senegal’s FP strategy focuses on community involvement and changing social norms* to increase contraceptive use, especially where fertility aligns with culture and religion. *Bajenu Gox (2009*) used respected women leaders as peer mentors during pregnancy^51^ and after birth, counselling women, men and family members, boosting antenatal, postnatal and FP referrals.^52^ *Religious involvement* was key: husbands’ groups were trained as FP champions,^53^ and the interfaith organisation promoted maternal health with Islamic birth spacing teachings. The Islam and Population Network provided faith-based FP guidance, showing Islam’s support for birth spacing. Religious leaders were trained to deliver messages and support outreach.

*The School for Husbands prom*oted male involvement in FP by educating men and encouraging shared decision-making with partners, positioning men as allies to transform gender dynamics. *Mass media and interpersonal communicatio*n promoted FP through campaigns in local languages, framing FP as birth spacing, aligned with Islamic values, rather than birth limitation. The 2014 Moytou Nef c*ampaign, supported by* religious leaders, promoted long birth intervals; its 2016 successor saw 68% of men discuss spacing and over 50% of individuals choose FP.^54^

**Figure 4:**
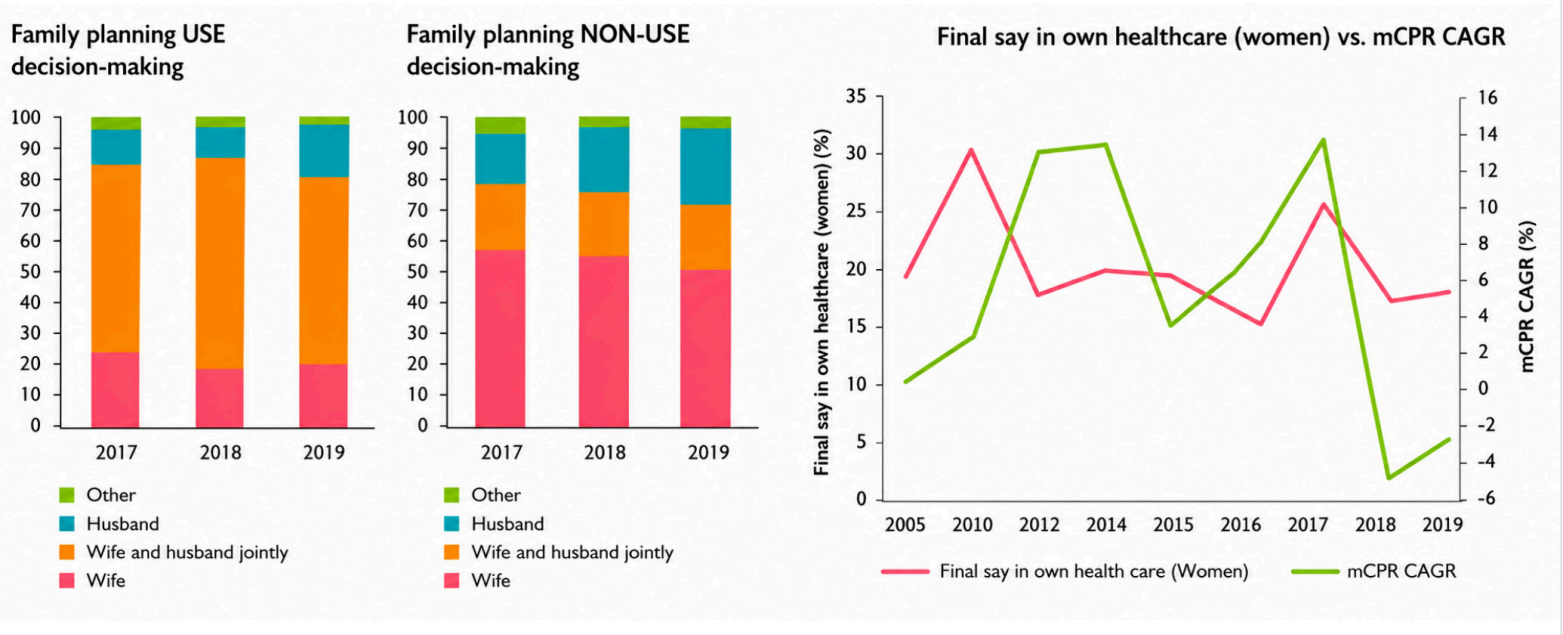
Gendered patterns in family planning decision-making and modern contraceptive use in Senegal. CAGR, compound annual growth raCompound Annual Growth Rate; Modern Contraceptive Prevalence RamCPR, modern contraceptive prevalence rate

Senegal engaged trusted figures like Bajenu Gox, religious leaders and husbands’ groups to promote FP within culturally acceptable narratives. These efforts helped legitimise FP, reshape norms and increase contraceptive knowledge.^55^ From 2005 to 2019, awareness of modern contraceptives among women aged 15–49 increased by 1.34 percentage points, accounting for 34.9% of the mCPR growth. Media campaigns most impacted women aged 21–30 years, while older women responded to fertility messages. Men’s exposure to FP messaging exceeded that of women from 2013 to 2017. This approach improved couple communication, with shared FP decisions rising from 43% in 2005 to 67% in 2019 (figure 4). As male involvement increased, women’s agency also improved. The rise in joint decision-making paralleled the growth of mCPR, underscoring the importance of gender-inclusive strategies.^56^ These findings show a complex FP process: women mostly decide alone not to use contraception, but decisions to use it are mainly made by men, with husbands acting as gatekeepers. The percentage of women reporting that their husbands make decisions alone increased by 131% from 2014 to 2019. This shift may reflect FP programmes targeting men as allies in reproductive health.^57^ While male engagement can boost contraceptive use and reduce opposition,^58^ increased male dominance in FP decisions raises concerns about reproductive autonomy if gender norms are unaddressed.^59 60^ Strategies that foster social approval, male involvement and intergenerational dialogue are all key to behaviour change.

#### Cross-sector interventions are essential drivers of FP progress

Senegal’s FP progress underscores the importance of cross-sector strategies like gender equality, education, empowerment and social norms in promoting informed reproductive choices, health equity and growth. Since 2012, FP has helped harness the demographic dividend, exemplified by policies like PSE. The 2015 launch of the National Gender Equality Strategy and Gender-Responsive Budgeting integrated reproductive health into governance. Education reforms and increased investment in girls’ education have improved FP outcomes, with girls’ primary school enrolment rising from 81.1% in 2000 to 91.2% in 2022, boosting their knowledge and attitudes about contraceptives.

Integrating FP with national development, gender budgeting and education strengthens women’s autonomy and decision-making. Decomposition analyses indicate that up to 18% of the growth in modern contraceptive use is due to increased knowledge. Income programmes targeting rural and low-income women have empowered them, enhancing decision-making and contraceptive negotiations within marriages. Increased knowledge and agency positively impact contraceptive use, highlighting the need for multisectoral policies addressing structural factors.

## Discussion and conclusion

Between 2010 and 2023, Senegal nearly doubled its mCPR from 12% to 25.6%, reflecting better access to modern methods and political support.^61 62^ Despite challenges like unmet youth needs, progress results from long-term, interconnected efforts. Combining strong leadership and coordinated actions with task-shifting, method diversification, community delivery and legal reforms shows how service delivery decentralisation and demand-generation strategies can accelerate FP progress and sustain gains. Broader reforms in gender, education and women’s empowerment bolster these efforts. These initiatives provide lessons for others seeking equitable, culturally responsive FP.

Government commitment involved legal reforms, the FP 2020 pledge, public financing and strategies like NFPAP. Multisectoral coordination and inclusive governance aligned donor and national investments. Despite relying on external funding, policy innovations improved contraceptive access. Political commitment created a supportive environment but did not directly boost FP use,^63^ similar to Ethiopia’s leadership and partnerships.^64^ As global health aid shifts towards local ownership, multilateral collaborations remain vital for country-led FP goals.^65^

Senegal’s rise in mCPR and demand fulfilment is attributed to health system improvements, including supply chain reforms, task-shifting and the expansion of community FP services. Initiatives such as the IPM, better access to injectables and implants and new service points have reduced stockouts and made affordable contraception accessible. Since the 1980s, the region has shifted towards demedicalising FP.^66^ CHWs, including Bajenu Gox and midwives, play a key role in providing contraceptives, increasing usage and supporting maternal care.^67^,^69^ Unlike Rwanda’s free door-to-door approach,^70^ Senegal’s facility-based strategy improves quality and equity through facility upgrades and training, adopting a rights-based model to broaden options and boost demand. Achieving health financing equity is vital for Universal Health Coverage in LMICs.^71^ Geographic access^72^ offers women more choices. Yet, a preference for injectables and implants raises concerns about provider bias and undermining comprehensive counselling.^73 74^ System pressures in Kenya and Ethiopia, driven by targets and donor priorities, have affected women’s autonomy^75^ and method diversity.^76^

To continue progress, Senegal should focus on informed choice, quality counselling and cross-sector investments that change social norms, rights-based, woman-centred reproductive care and promote contraceptive use. Senegal effectively engages with religious leaders and tailors its efforts to local contexts. Behaviour change programmes promoting positive masculinity and open spousal communication have shifted, but not fully balanced, the couple’s power dynamics. Studies show that involving family members like mothers-in-law increases contraceptive use.^77 78^ A Niger programme for husbands improved communication, gender attitudes and contraception support.^79^ Male involvement is vital, but focusing only on awareness or gatekeeping is ineffective.^80 81^ Gender-transformative approaches often lack intersectional analysis and may reinforce male control.^59 60^ Programmes tend to overlook younger men, unmarried partners and rural or conservative groups, despite their influence.^82^ Progress needs shared decision-making, equitable partnerships^83^ and strategies that challenge norms, promote respect and support joint reproductive planning—especially among youth.^84 85^ Women’s education and economic opportunities increase their agency, improving FP decisions.^86 87^ Education links to greater FP knowledge, decision-making and contraceptive use.^88^,^90^

Since 2010, investments in Senegal haven't increased mCPR beyond 2017 levels. Early gains focused on urban, educated, married women, but expanding to adolescents, unmarried women and rural areas faces barriers like stigma and limited autonomy. mCPR’s failure to meet unmet needs and improve care quality may contribute to its stagnation. Supply efforts, such as task-shifting and offering additional methods, continue; however, issues—method bias, training gaps, stockouts—persist.^91^ Community distribution has improved access but remains uneven, especially for adolescents.^92 93^ Youth outreach lag, risking demand decline due to lower investments and stagnant external funding.^94 95^ Addressing these challenges requires equity-focused, context-sensitive strategies based on lessons from other countries. In India, mCPR improvements have been driven by CHWs, integration of FP into broader health services and gender-responsive and youth-responsive programming.^96 97^ Malawi expanded rural access by authorising Health Surveillance Assistants to deliver injectables.^98^ Kenya successfully integrated FP with HIV/AIDS services while engaging youth, men and community leaders to overcome sociocultural barriers.^90 91 96 97^ Ethiopia increased mCPR from 14% to 41% (2005–2019) through its Health Extension Program, delivering FP services to rural households.^99 100^ Rwanda’s rise in mCPR—from 17% in 2005 to 52% by 2020—was driven by political leadership, CHWs and demand-generation campaigns.^101^ Senegal’s progress depends on external aid and faces gaps in adolescent services and inconsistent efforts. Donor dependence causes fragmented initiatives and vulnerability. To advance, Senegal should expand successful, culturally sensitive, gender-transformative and adolescent-focused models, increase domestic funding and attract cross-sector investments.^102^

### Study limitations

Limitations include data gaps during strikes, focus on successful policies, mixed results from different data types and the complexity of mixed-methods research. These factors affect trend accuracy and insights into underperforming programmes, requiring careful interpretation despite improved reliability from triangulation.

## Supplementary material

10.1136/bmjgh-2024-018774 online supplemental table 1

10.1136/bmjgh-2024-018774 online supplemental table 2

10.1136/bmjgh-2024-018774 online supplemental file 1

10.1136/bmjgh-2024-018774 online supplemental file 2

## Data Availability

All data relevant to the study are included in the article or uploaded as supplementary information.
