# Supplementary material for: Policy in practice: assessing Senegal’s family planning progress using a mixed-methods approach
Source: BMJ Glob Health. 2026 Jun 9;11(Suppl 3):e018774. doi: 10.1136/bmjgh-2024-018774 (PMC13250212; doi:10.1136/bmjgh-2024-018774)
Supplement: online supplemental table 1 [file bmjgh-11-Suppl_3-s001.pdf]

Supplemental Table 1. PRISMA 2020 Flowchart summarizing study selection for the systematic review of Family Planning policies and outcomes in Senegal (2000–2022)

Caption: This flowchart illustrates the study selection process for the systematic review, adhering to PRISMA 2020 guidelines. It displays the number of records identified from each database, after duplicates are removed, the number of studies screened, the number of full-text articles assessed for eligibility, and the final number of studies included. Reasons for exclusion at the full-text stage (e.g., unrelated topic, editorial, or insufficient data) are also indicated. Data sources: PubMed, Web of Science, grey literature, and reference lists of relevant studies published between January 2000 and December 2022.

[Identification]

| Records identified through database searching (n = 850) |
| - PubMed: 500 |
| - Web of Science: 350 |
| Additional records identified through other sources (n = 50) |
| - Reference lists / grey literature: 50 |

|

▼

[Duplicates Removed]

| Records after duplicates removed (n = 780) |

|

▼

[Screening]

| Records screened (titles & abstracts) (n = 780) |
| Records excluded (n = 600) |
| - Not FP-related: 250 |
| - Not Senegal-focused: 150|
| - Outside date range: 100 |
| - Editorial/commentary: 100 |

|

▼

[Eligibility]

| Full-text articles assessed for eligibility (n = 180) |
| Full-text articles excluded (n = 120) |

| - No FP outcomes: 50 |
| - No policy/financing data: 40 |
| - Duplicate publication: 20 |
| - Other reasons: 10 |

|
▼

[Included Studies]

| Studies included in qualitative synthesis (n = 60) |
| Studies included in quantitative synthesis (meta-analysis) (n = 40) |
