# Supplementary material for: Policy in practice: assessing Senegal’s family planning progress using a mixed-methods approach
Source: BMJ Glob Health. 2026 Jun 9;11(Suppl 3):e018774. doi: 10.1136/bmjgh-2024-018774 (PMC13250212; doi:10.1136/bmjgh-2024-018774)
Supplement: online supplemental table 2 [file bmjgh-11-Suppl_3-s002.docx]

**Supplemental Table 2 : Timeline of Key Family Planning Policies and Programs in Senegal**

**Caption:**This table summarizes the evolution of major family planning (FP) policies, strategies, and programs in Senegal over time, highlighting their objectives, target populations, and implementation periods. The timeline includes national initiatives, donor-supported programs, and policy reforms that have influenced contraceptive access, availability, and uptake across the country. Data sources: Ministry of Health and Social Action (MoHSA) reports, DHS policy reviews, and peer-reviewed literature (2000–2022).

| **Year(s)** | **Policy/Program** | **Type** | **Objective/Impact** | **mCPR (%)** |
| --- | --- | --- | --- | --- |
| **1980** | **Law No. 80-49**  MoHSA, (UNFPA). | FP Policy Statement | Legalized contraception; allowed FP service delivery through public and NGO sectors. | — |
| **1988** | **Population Policy Declaration**  Ministry of Economy, Finance. | National population policy | Official government endorsement of FP to reduce population growth. | — |
| **1990** | **National FP Program** (MoHSA, UNFPA, ASBEF) | National FP Program | Promote voluntary FP, birth spacing; reduce maternal/infant mortality; strengthen health systems. | 4.8 |
| **1997** | **FP Policy Statement**  (MoHSA, CNP, partners) | FP Policy Statement | Ensure access to modern FP; support youth, decentralization, and supply chains. | 8.1 |
| **1998–2000** | **Fiscal Reform FP Products**  WAEMU, MoHSA | Regulatory and financing policy | Removed import duties on FP products; introduced VAT; improved affordability and logistics. | — |
| **2002** | **National Population Policy**  CNP, Ministry of Economy and Finance, MoHSA, UNFPA | National population policy | Link FP to sustainable development; empower women/youth; improve multisectoral coordination. | — |
| **2003** | **Decree FP Product Pricing**  MoHSA, Ministry of Finance | Regulatory and financing policy | Capped retail margins for contraceptives in the public sector. | — |
| **2005–2006** | **Reproductive Health Law**  MoHSA, UNFPA, WHO, USAID | National FP Program | Guaranteed rights to RH services; promoted informed choice and gender equity. | 10.3 |
| **2006** | **Community Health Program**  MoHSA, Child Fund | National policy framework | Revitalized rural health huts; expanded CHWs; integrated maternal and child health. | — |
| **2006–2011** | **Task Shifting Initiative**  MoHSA, UNFPA, USAID, IntraHealth International | service delivery innovation | Authorized CHWs/mid-level providers to deliver FP (e.g., pills, IUDs, injectables). | — |
| **2009–2018** | **National Health Development Plan (PNDS)**  MoHSA WHO, World Bank | National policy framework | Strategic framework aligned with MDGs; promoted community partnership in service delivery. | 12.1 |
| **2009** | **Bajenu Gox Initiative**  MoHSA | Community engagement demand-generation | Culturally respected women promoted FP and MNCH through community engagement. | — |
| **2010–2011** | **Ouagadougou Call to Action** | FP Policy Statement | Regional commitment to reach 27% mCPR by 2015. | — |
| 2010-2011 | **Long-Acting Reversible Contraceptives**  MoHSA, UNFPA, USAID | Service delivery / Method mix expansion | diversify contraceptive options and expand access; supported task-shifting to allow CHWs to provide injectables. |  |
| **2010–2015** | **Urban Health Initiative (ISSU)**  MoHSA, IntraHealth | Service delivery and demand-generation | Boosted FP demand and quality; free services for the urban poor; introduced Informed Push Model (IPM). | 17 → 22 |
| **2011–2012** | **IPM Pilot**  MoHSA | supply chain management innovation | Replaced “pull” system with a monthly supply push model using trained logisticians. improve availability across all levels of public health system, | — |
| **2013–2016** | **National IPM Expansion** | supply chain management innovation | Scaled IPM nationwide; improved stock availability and logistics. | 12 → 26.3 |
| **2012** | **London Summit on FP** | FP Policy Statement | National FP Action Plan pledged; targeted 27% mCPR by 2015. | 16.1 |
| **2012** | **Reproductive Health and Child Survival Directorate** | Program management unit | Enhanced RH service coordination, data systems, and task shifting. | — |
| **2012** | **NAPFP 3D Model**  MoHSA | National FP Program | Strengthened supply chain and community engagement; integrated FP with primary care. | — |
| **2012** | **School of Husbands**  Ministry of Women, MoHSA | Community engagement and demand-generation | Engaged men to support RH; improved gender relations and community mobilization. | — |
| **2013** | **Moytou Nef Campaign** | Community engagement and demand-generation | Mobile services for remote areas; religious leader engagement. | — |
| **2014** | **National Strategic Plan for Community Health (NSPCH)** | National policy framework | Strengthened CHWs and coordination between community and health systems. | — |
| **2014–2015** | **Monthly Special FP Days**  MoHSA, TCI, IntraHealth International | Community engagement,demand-generation | Outreach to underserved and youth through CHW-led services. | — |
| **2016** | **RRHCSD becomes**  **Directorate of Maternal and Child Health (DSME)** | Program management unit | FP integrated within broader MNCH agenda; aligned with FP2020 goals. | 23.1 |
| **2016–2020** | **FP Strategic Framework** | National FP Program | Renewed commitment to equity, public-private services, and system reform. integrating gender-responsive programming. adolescent-friendly services, | — |
| **2016–2017** | **LARCs and injectables Expansion** | Delivery/ Method mix expansion | Scaled long-acting methods and self-injection; delivery through all sectors including CHWs. | 26.3 |
| **2017-2018** | **Transfer of IPM management to PNA** | contraceptive supply chain | National Supply Agency (PNA) took over contraceptive supply management. | 25.4 |
| **2019** | **Task shifting and community health worker (CHW) role expansion** | Health systems strengthening service delivery | Further authorized CHWs to provide injectables and counseling, increasing FP access in rural areas. | 25.5 |
| **2020** | **Adaptation of FP services amid COVID-19** | Health systems strengthening service delivery | Ensured continuity through increased self-injection adoption and use of digital platforms for FP demand generation and client follow-up. | — |
| **2022** | **Digital health innovations in FP supply and monitoring** | supply chain management innovation | Adoption of digital logistics management information systems improved real-time contraceptive stock tracking and data-driven decision-making at all health system levels. | 25.6 |
