# Supplementary material for: Policy in practice: assessing Senegal’s family planning progress using a mixed-methods approach
Source: BMJ Glob Health. 2026 Jun 9;11(Suppl 3):e018774. doi: 10.1136/bmjgh-2024-018774 (PMC13250212; doi:10.1136/bmjgh-2024-018774)
Supplement: online supplemental file 1 [file bmjgh-11-Suppl_3-s003.pdf]

BMJ Global Health Author Reflexivity Statement

Adapted from Morton, B., Vercueil, A., Masekela, R., Heinz, E., Reimer, L., Saleh, S., Kalinga, C., Seekles, M., Biccard, B., Chakaya, J., Abimbola, S., Obasi, A. and Oriyo, N. (2022), Consensus statement on measures to promote equitable authorship in the publication of research from international partnerships. Anaesthesia, 77: 264-276. https://doi.org/10.1111/anae.15597

Author Reflexivity Statement – Policy in Practice: Assessing Senegal's Family Planning Progress Using a Mixed-Methods Approach

**Study conceptualisation**	
1. How does this study address local research and policy priorities?	The study responds directly to Senegal's national priority of strengthening family planning (FP) within its reproductive, maternal, newborn, and child health (RMNCH) agenda. It aligns with the Ministry of Health and Social Action's (MoHSA) Family Planning Investment Plan and contributes evidence for performance reviews under the Plan National de Développement Sanitaire et Social (PNDSS). The research was initiated through the Directorate of Reproductive and Maternal collaboration, aimed at generating actionable, context-specific insights to improve FP access, equity, and financing
2. How were local researchers involved in study design?	The study was co-designed by researchers from Université Cheikh Anta Diop (UCAD), MoHSA, and international partners (Aga Khan University and The Hospital for Sick Children). Senegalese investigators led the identification of key research questions, contextualized data sources, and adapted the WHO health systems framework to national priorities. A participatory design process ensured integration of local policy perspectives and FP programmatic realities.
**Research management**	
3. How has funding been used to support the local research team(s)?	Funding supported national research activities, including local data collection, stakeholder consultations, and policy review workshops. Resources were allocated to strengthen national analytic capacity, particularly at UCAD and the MoHSA's Directorate of Mother and Child Health.
**Data acquisition and analysis**	
4. How are research staff who conducted data collection acknowledged?	Local research assistants, community health workers, and MoHSA focal points who facilitated data access and fieldwork are co-authors in the paper. Their institutional contributions are formally recognized.
5. How have members of the research partnership been provided with access to study data?	All data were collected, owned, and stored by Senegalese institutions. Shared data files and analytic templates were jointly reviewed by national and international partners. Data analysis was led by UCAD researchers with technical support from international collaborators.
6. How were data used to develop analytical skills within the partnership?	National researchers received hands-on training in decomposition analysis and policy synthesis through joint workshops and mentorship. Skills in using DHS datasets, thematic synthesis, and mixed-method integration were strengthened.

**Data interpretation**	
7. How have research partners collaborated in interpreting study data?	Joint data interpretation sessions were held with MoHSA, UCAD, and international experts to triangulate quantitative and qualitative findings. A convergence matrix was used to compare evidence streams, and results were validated through stakeholder consultations.
**Drafting and revising for intellectual content**	
8. How were research partners supported to develop writing skills?	The first draft was prepared by Senegalese authors, who received mentorship in structuring manuscripts for international peer-reviewed journals. Writing workshops were conducted to strengthen analytical narrative and alignment with journal standards.
9. How will research products be shared to address local needs?	Findings will be disseminated through national policy briefs, workshops with MoHSA and regional health authorities, and presentations at the FP Technical Working Group. A French-language summary will be shared with community stakeholders and journalists to foster evidence uptake.
**Authorship**	
10. How is the leadership, contribution and ownership of this work by LMIC researchers recognised within the authorship?	Senegalese researchers are lead and corresponding authors. Authorship order reflects substantive intellectual contribution, with local leadership prioritized at all stages—from design to interpretation and drafting.
11. How have early career researchers across the partnership been included within the authorship team?	Early-career Senegalese researchers participated in data coding, analysis, and manuscript drafting. Their contributions were mentored by senior national and international investigators and recognized through co-authorship. They are co-authors
12. How has gender balance been addressed within the authorship?	Gender balance was actively considered: over half of the core authorship team are women, including lead analysts and qualitative researchers. Leadership roles were distributed without gender bias.
**Training**	
13. How has the project contributed to training of LMIC researchers?	The project contributed to building technical and writing capacity in FP data analysis, decomposition methods. UCAD researchers received mentorship in research dissemination and policy engagement.
**Infrastructure**	
14. How has the project contributed to improvements in local infrastructure?	The project strengthened analytic infrastructure through the creation of data-sharing protocols and a national FP evidence dashboard to inform ongoing policy review.
**Governance**	
15. What safeguarding procedures were used to protect local study participants and researchers?	All qualitative interviews followed ethical guidelines approved by the Senegalese National Ethics Committee. Data were anonymized and stored securely. Research teams were trained on confidentiality and participant protection.
