# Supplementary material for: Policy in practice: assessing Senegal’s family planning progress using a mixed-methods approach
Source: BMJ Glob Health. 2026 Jun 9;11(Suppl 3):e018774. doi: 10.1136/bmjgh-2024-018774 (PMC13250212; doi:10.1136/bmjgh-2024-018774)
Supplement: online supplemental file 2 [file bmjgh-11-Suppl_3-s004.pdf]

Policy in practice: Assessing Senegal's Family Planning progress using a mixed-method approach

Sylvain Landry Birane FAYE(1), Georgette Helene Coumba SOW(2), Rose André FAYE(1), Marie Gabrielle NDONG(1), Martine Eva Tine(1,3), Ndeye Awa Diagne(3), Amadou Doucoure(3), Hina Najmi(4), Mishal Zulfiqar(4), Sacha St-Onge Ahmad(5), Zahid Ali Memon(6) and Zulfiqar Bhutta(5)

[1]Laboratoire de Sociologie, d'Anthropologie et de Psychologie (LASAP- ETHOS) FLSH, Université Cheikh Anta DIOP – DAKAR – SENEGAL.
[2] S&F Pro Consulting LLC – Manteca, California – USA
[3] Ministry of Health and Social Action, Mother and Child Health Directorate, SENEGAL
[4] Institute of Global Health and Development, Aga Khan University
[5] The Hospital for Sick Children, (Sickkids) Toronto, Canada
[6] Community Health Sciences Department, Aga Khan University

Corresponding author: fayesylvain@yahoo.fr

ABSTRACT

Background: Senegal has made significant progress in family planning (FP), with the modern contraceptive prevalence rate (mCPR) increasing from 12% in 2010 to 26% in 2023, along with a sharp decline in unmet need. Once among West Africa's lowest performers, Senegal now stands out for its advancements in FP access and equity.

Objectives: This study examines the main factors driving Senegal's FP progress—including policy leadership, financing mechanisms, and community engagement—while identifying ongoing challenges and lessons for policy and practice.

Methods: We employed a mixed-methods approach, combining trend and decomposition analyses with a review of FP policies, financing, and programs, guided by the WHO's health system building blocks. Qualitative data were collected from interviews and focus groups with policymakers, providers, community leaders, and users across various regions.

Results: Senegal's early gains in FP were driven by strong political leadership, task-shifting, community-based service delivery, and supply chain improvements. Social mobilization campaigns, including *Moytou Nef*, and engagement with religious leaders and men promoted supportive norms and increased demand. These efforts improved method availability, reduced stockouts, and expanded access. Decomposition analysis identified FP knowledge (35%), education (18%), and facility access (16%), particularly among young women, as the main drivers of performance. Broader shifts in gender norms, women's empowerment, and male involvement also supported uptake. Since 2017, mCPR has plateaued, with persistent gaps among adolescents, unmarried women, and rural populations due to stigma, provider bias, limited youth-friendly services, and donor dependence. These findings highlight the need for targeted, equity-focused interventions to sustain and expand Senegal's FP achievements.

Conclusion: Senegal's FP progress demonstrates how strategic governance, service innovation, and community engagement can increase contraceptive use. To sustain and build on these achievements, renewed investment is necessary in youth-focused services, domestic funding, and equity-centered strategies that are integral to larger health and development plans.

Keywords: Family planning, contraceptive use, mCPR, Health System Strengthening, Senegal, Community-Based Interventions, Gender and Youth Equity, Task-Shifting, Policy and Governance Reform

KEY MESSAGES

What is already known about this topic

- Senegal is recognized as a rare family planning (FP) success story in West Africa, with modern contraceptive use rising steadily from a low baseline.
- Most prior studies have focused on donor-funded programs and their outcomes, overlooking the role of sustained national leadership, strong governance, and coordinated policy action in enabling this progress.
- Despite clear gains, deep and persistent inequities—especially among adolescents, rural women, and conservative communities—continue to threaten equitable FP access.

What this study adds

- Provides the first comprehensive analysis of Senegal's FP progress, revealing how deliberate policy sequencing, political consistency, and innovation in service delivery and supply systems sustained long-term improvements.
- It demonstrates that institutional continuity, adaptive governance, and community-based models were crucial in expanding access and resilience across regions.
- Demonstrates that gender-transformative action—not male engagement alone—is essential to advance women's autonomy and tackle entrenched social norms limiting FP use.

How this study may affect research, practice, or policy

- Offers a replicable mixed-methods framework for assessing reproductive health progress in low- and middle-income settings.
- Strengthens the case for embedding FP within broader health system reforms, with sustained investment in trained community health workers and decentralized services.
- Calls for a shift from short-term awareness campaigns to long-term, culturally grounded norm-change strategies led by trusted community actors and backed by political and financial commitment.
